# The impact of a multicomponent telemedicine-based intervention on quality of life in adults with respiratory failure requiring mechanical ventilation: protocol for a cluster stepped-wedge randomized clinical trial (Tele-Rehab MV Trial)

**DOI:** 10.62675/2965-2774.20250136

**Published:** 2025-11-27

**Authors:** Adriano José Pereira, Rafael Barberena Moraes, Geraldine Trott, Maura Cristina dos Santos, Duane Mocellin, Alessandra Yuri Takehana de Andrade, Aline Paula Miozzo, Luisa de Castro Miranda Paixão, Raíne Fogliati de Carli Schardosin, Carla Luciana Batista, Emelyn de Souza Roldão, Cilene Saghabi de Medeiros Silva, Rosa da Rosa Minho dos Santos, Maria Isabel Costa e Silva Cavalcanti, Jennifer Menna Barreto de Souza, Luciana Diniz Nagem Janot de Matos, Denise de Souza, Juliana Wanderley Cidreira Neves, Gabriela Soares Rech, Thais Martins de Almeida Souza, Gabrielle Nunes da Silva, Carolina Rothmann Itaqui, Silvana Maria Silva Yoshida, Raquel Afonso Caserta Eid, Marcio Luiz Ferreira de Camillis, Kamilla Silvestre Rahman Genena, Leonardo Miguel Correa Garcia, Ester Cavalcanti Schaefer, Priscila Alves Pereira Cidade, Nara Fabiana Mariano, Isadora Rebolho Sisto, Ana Cristina Lagoeiro Patrocinio da Cruz, Camille Lacerda Corrêa, Ivan Ramos Maia, Juliana de Oliveira, Andrea de Carvalho, Marcio Ramos Laguna, Leonardo Rolim Ferraz, Cassiano Teixeira, Yasmin Ferreira Cavaliere, Fernando Godinho Zampieri, Regis Goulart Rosa

**Affiliations:** 1 Hospital Israelita Albert Einstein São Paulo SP Brazil Hospital Israelita Albert Einstein - São Paulo (SP), Brazil.; 2 Hospital Moinhos de Vento Porto Alegre RS Brazil Hospital Moinhos de Vento - Porto Alegre (RS), Brazil.; 3 Universidade Federal do Rio Grande do Sul Porto Alegre RS Brazil Postgraduate Program in Pneumological Sciences, Universidade Federal do Rio Grande do Sul - Porto Alegre (RS), Brazil.

**Keywords:** Respiration, artificial, Critically illness, Patient discharge, Patient readmission, Respiratory insufficiency, Rehabilitation, Quality of life, Telemedicine, Quality improvement, Interview

## Abstract

**Objective:**

To assess the impact of a multicomponent intervention on the health-related quality of life of patients with hypoxemic respiratory failure requiring invasive mechanical ventilation.

**Methods:**

A cluster stepped-wedge randomized clinical trial will be conducted in intensive care units across Brazil. Intensive care units with ≥ 8 beds and the capacity to admit patients with acute hypoxemic respiratory failure will be included. Within each intensive care unit, adult patients with acute hypoxemic respiratory failure requiring invasive mechanical ventilation, in whom SARS-CoV-2 infection is part of the differential diagnosis, will be enrolled. The intervention consists of a telemedicine-based quality improvement program focused on disability prevention and rehabilitation strategies, implemented during the patient's intensive care unit stay, continued through ward admission, and extending up to 2 months post-hospital discharge. The primary outcome is health-related quality of life assessed using the EuroQol 5-Dimension 3-Level scale 90 days after discharge from the hospital. Secondary outcomes include rehospitalization within 30 days from hospital discharge, as well as all-cause mortality, anxiety, depression, cognitive impairment, new disabilities for instrumental activities of daily living, and return to work or studies 90 days after discharge from the hospital.

**Results:**

The study protocol has been approved by the research ethics committees of all participant institutions. It was registered at ClinicalTrials.gov (NCT06343545) before the first participant was included. We aim to disseminate the findings through conferences and peer-reviewed journals.

**Conclusion:**

The "Tele-Rehab MV trial" may provide further information on the role of early multicomponent interventions aimed at disability prevention and rehabilitation for critically ill patients with acute hypoxemic respiratory failure.

## INTRODUCTION

Acute respiratory failure requiring invasive mechanical ventilation (MV) is associated with significant mortality rates.^(1,2)^ Furthermore, survivors often develop new physical, mental, and cognitive disabilities, as well as worsening clinical conditions, which can significantly impair their health-related quality of life.^(3–5)^ Coronavirus disease 2019 (COVID-19) remains a notable cause of acute respiratory failure and long-term disabilities,^(6)^ with the added potential to cause persistent symptoms regardless of the infection's initial severity.^(7,8)^ Although the incidence of severe acute respiratory syndrome coronavirus 2 (SARS-CoV-2)-related respiratory failure has declined with the widespread adoption of vaccination, it persists as an endemic cause of pneumonia, particularly among patients with significant comorbidities,^(9)^ and is often part of the differential diagnosis in severe cases of acute hypoxemic respiratory failure.^(10)^ While bundles of interventions, including analgesia optimization, sedation minimization, early mobilization, and delirium prevention, as well as screening for individuals at risk of new disabilities for early rehabilitation, have been recommended to prevent disabilities in critical care patients,^(4,11,12)^ no large randomized clinical trial has yet demonstrated a significant impact on long-term health-related quality of life.^(3,12)^ Additionally, the burden of disability following critical illness is often associated with patients’ inability to attend clinic-based follow-up, and telemedicine may serve as a tool to reduce healthcare inequalities.^(13)^ Accordingly, the primary objective of this study is to assess the impact of a multicomponent intervention on the health-related quality of life of patients with hypoxemic respiratory failure requiring invasive MV. We hypothesize that compared to the standard of care, the intervention will improve the quality of life of patients with acute hypoxemic respiratory failure requiring invasive MV.

## METHODS

### Study design

**Figure 1:**
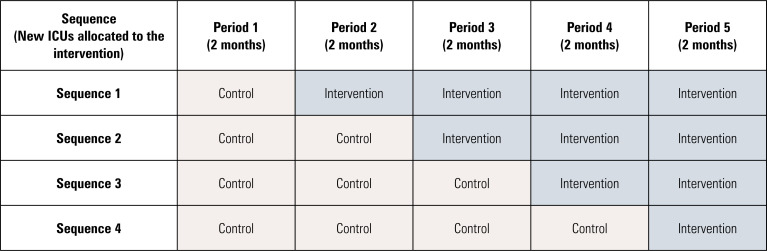
Study design.

This study is a cluster stepped-wedge randomized clinical trial, with the intensive care unit (ICU) as the unit of randomization. The study begins with an initial control period during which none of the ICUs are exposed to the intervention. Subsequently, at regular 2-month intervals (the "steps"), a randomly selected group of ICUs transitions from the control to the intervention. This sequential process continues until all ICUs have crossed over and are fully exposed to the intervention (Figure 1). This design was chosen because the intervention targets organizational-level components and is intended to be implemented across the entire ICU rather than in selected patients. The cluster stepped-wedge design helps mitigate the risk of contamination between groups and accommodates the logistical challenges of a sequential roll-out across participating centers.

ICU - intensive care unit.

Randomization will be stratified based on the number of ICU beds (≤ or > median) to minimize numerical imbalance between study arms due to clusters with differing care capacities. A statistician not involved in study implementation or recruitment will generate the randomization list, thereby ensuring allocation concealment and minimizing potential bias. The list will be stored as a digital file in a secure, password-protected electronic system accessible only to the statistician. Intensive care unit allocation will remain concealed from all study investigators. It will be disclosed by the coordination center to the local research team 60 days prior to implementation to allow adequate time for staff training. Local research teams will be instructed to maintain usual care practices until the official start date of the intervention period. Due to the nature of the interventions and study design, blinding participants or ICU staff is not feasible. However, outcome evaluators will remain blinded.

The present study protocol follows the Standard Protocol Items: Recommendations for Interventional Trials statement recommendations (SPIRIT).^(14)^ This study protocol was registered at Clinicaltrials.gov (NCT06343545).

### Participants

#### Cluster eligibility criteria

Brazilian adult ICUs of public hospitals with at least eight beds, capable of admitting cases of respiratory failure with suspected COVID-19, capable of doing remote care (internet link available in the hospital), and able to provide institutional agreement to participate in the study will be enrolled. All units will be considered part of the same cluster in hospitals with multiple ICUs. Participating centers will be selected based on the number of reported cases of severe acute respiratory infection during the year preceding the study, as provided by the Secretariat for Health and Environmental Surveillance of the Brazilian Ministry of Health. In each Brazilian state, the public hospital with the most notifications will be invited via email. Invitations will be sent consecutively until the required number of clusters is reached.

#### Patient eligibility criteria

Consecutive patients aged 18 years admitted to the ICU with hypoxemic acute respiratory failure requiring invasive MV, in whom SARS-CoV-2 infection is part of the differential, will be enrolled. This does not imply that COVID-19 is the primary suspected cause, but rather that it is considered at least a possible (though not necessarily probable) diagnosis at the time of ICU admission. The exclusion criteria are as follows: severe underlying disease with a life expectancy of ≤ 3 months, as judged by the local treating physician; absence of a responsible family member in cases where patients have communication barriers (e.g., aphasia, severe cognitive impairment, or non-native Portuguese speakers); lack of telephone contact; and unavailability for telephone follow-ups. Participants will be enrolled only once and will not re-enter the study.

Importantly, a confirmatory test for SARS-CoV-2 infection will not be required for inclusion. Even if an alternative diagnosis (e.g., bacterial pneumonia, pulmonary embolism, or pulmonary edema) is confirmed during follow-up, the patient will remain in the study, given that the intervention may benefit all patients with acute hypoxemic respiratory failure, regardless of the underlying etiology.^(5)^

### Interventions

The study intervention is an evidence-based,^(4,15–18)^ multicomponent program designed to prevent disability and promote rehabilitation through telemedicine. It was developed through a consensus process involving a multidisciplinary board of critical care clinicians and rehabilitation specialists (physiatrists, respiratory and motor therapists, psychologists, and speech-language therapists) explicitly assembled for this study. The program is structured into three principal telemedicine-based bundles: the ICU tele-bundle, the ward tele-bundle, and the post-discharge tele-bundle (Table 1). The ICU tele-bundle is implemented by local clinical staff following training and is reviewed remotely by a study critical care clinician through three 1-hour telemedicine sessions per week. For each patient, the bundle is discussed during daily multidisciplinary rounds and includes strategies to optimize analgesia, minimize sedation exposure, conduct spontaneous breathing trials systematically, implement delirium prevention measures, promote early mobilization, and remove unnecessary invasive devices. The ward tele-bundle, delivered either by local clinical staff or centralized tele-rehabilitation specialists from the coordination center, aims to identify patients at high risk for post-ICU complications, including those with nutritional impairment, oropharyngeal dysphagia, low physical capacity, and anxiety or depression symptoms using validated screening tools.^(19–22)^ It also involves developing a personalized tele-rehabilitation plan to be initiated during ward hospitalization (e.g., nutritional guidance, physical and respiratory therapy, swallowing rehabilitation, and psychological follow-up) and continued after hospital discharge. Lastly, the post-discharge tele-bundle comprises a 2-month centralized tele-rehabilitation program, delivered via smartphone videoconferencing, and coordinated by the central study team. This program includes nutritional guidance, swallowing rehabilitation, physical therapy, respiratory therapy, and psychological support, building upon the individualized rehabilitation plan developed during the ward phase. To ensure continuity of care, dedicated nurse navigators will engage in multidisciplinary discussions via telemedicine with ICU staff, ward staff, and tele-rehabilitation specialists, addressing challenges and providing ongoing guidance throughout the intervention phases.

**Table 1:** Components and rationale of study intervention

Intervention	Components	Delivery*	Rationale
**ICU tele-bundle**			
Analgesia optimization	– Use of scales to assess patient-reported pain, such as the numerical rating scale and behavioral pain scale – Use of intravenous opioids as the first-line drug class for non-neuropathic pain – Use of a multimodal pharmacological analgesia approach – Use of nonpharmacological interventions such as relaxation techniques, massage, and music therapy – Preemptive analgesia before potentially painful procedures	– Local ICU staff, with support from discussions during daily multidisciplinary rounds	– Ensuring adequate pain control improves patient comfort, reduces stress, and may help reduce long-term impairments, such as symptoms of anxiety, depression, post-traumatic stress, and delirium-related cognitive dysfunction
Minimization of sedation	– Prioritization of analgesia before administering sedative agents – Use of scales to assess the level of sedation, such as RASS – Titration of sedative agents to a light sedation (e.g., RASS −2 to +1) unless contraindicated (e.g., intracranial hypertension, status epilepticus, and use of neuromuscular blocking agents) – Avoidance of benzodiazepines as the first-line drug class for sedation	– Local ICU staff, with support from discussions during daily multidisciplinary rounds	– Light sedation is associated with better short-term outcomes, including higher survival rates, more ventilator- and ICU-free days, and fewer incidences of delirium, muscle weakness, and symptoms of depression and post-traumatic stress. Minimizing sedation may improve long-term physical function, reduce delirium-related cognitive dysfunction, and promote better mental health
Spontaneous breathing trials	– Daily Screening: Candidates for SBT are identified by the absence of hypoxia, apnea, agitation, significant vasopressor use, or elevated intracranial pressure – SBT Procedure: Trials lasting 30 - 120 minutes are conducted with a T-piece or Pressure Support to evaluate readiness for independent breathing – Outcomes: Successful trials, indicated by the absence of tachypnea, hypoxia, diaphoresis, or instability, suggest readiness for extubation. Failed trials, marked by respiratory fatigue or desaturation, lead to termination and resumption of ventilatory support	– Local ICU staff, with support from discussions during daily multidisciplinary rounds	– SBTs assess readiness for extubation, reducing risks of prolonged mechanical ventilation, such as exposure to sedatives, muscle weakness, and delirium. SBTs may contribute to improved long-term physical health and cognitive function
*Delirium* prevention	– Ensuring temporal and spatial orientation – Preventing sensory deprivation by allowing the use of glasses or hearing aids when indicated – Promoting an adequate sleep-wake cycle by minimizing nighttime interruptions and maximizing daytime light exposure when possible – Avoiding benzodiazepine use whenever possible – Enhancing communication for mechanically ventilated patients through writing, communication boards, or phonatory cannulas for tracheostomized patients	– Local ICU staff, with support from discussions during daily multidisciplinary rounds	– Delirium is associated with long-term cognitive dysfunction and post-traumatic stress, and its prevention helps reduce these outcomes
Early mobilization	– Daily assessment of mobility feasibility and safety, considering contraindications such as hemodynamic instability, prone positioning, uncontrolled intracranial hypertension or seizures, unstable spinal cord injury, or RASS > +2 – Early initiation of physical activity, preferably within 48 hours of ICU admission. – Mobilization strategies based on clinical condition, including verticalization, bedside sitting, trunk control exercises, and out-of-bed mobilization – Session duration adjusted to patient stability and endurance to prevent fatigue	– Local ICU staff, with support from discussions during daily multidisciplinary rounds	– Early mobilization preserves muscle strength, reduces the risk of complications like pneumonia and deep vein thrombosis, shortens ICU stays, and is associated with lower occurrences of muscle weakness at ICU discharge and long-term cognitive dysfunction
Removal of unnecessary invasive devices	– Daily assessment of the necessity of maintaining invasive devices, such as central venous catheters, arterial lines, and urinary catheters	– Local ICU staff, with support from discussions during daily multidisciplinary rounds	– Early removal of non-essential devices reduces the risk of infections, enhances patient comfort, facilitates mobilization, minimizes delirium, and accelerates recovery, potentially improving long-term cognitive function and physical health
**Ward tele-bundle**	– Screening✝ patients at high risk for post-ICU complications – Nutritional impairment: according to the NRS 2002 – Oropharyngeal dysphagia: according to the EAT-10, or requirement of tracheostomy, or length of mechanical ventilation >48 hours – Low physical capacity: according to the modified Barthel Index – Anxiety or depression symptoms: according to the HADS – Creation of a tailored rehabilitation plan (based on the screening) to be initiated during ward hospitalization and continued after hospital discharge – Nutritional guidance – Swallowing rehabilitation – Physical therapy – Respiratory therapy – Psychological follow-up	– Local ward staff and rehabilitation specialists via centralized tele-rehabilitation through smartphone videoconferencing	– Screening patients at high risk for post-ICU complications enables early identification and intervention to prevent or mitigate long-term issues such as physical disability, cognitive dysfunction, and psychological problems. Creating a tailored rehabilitation plan based on this screening ensures targeted support to optimize long-term recovery
**Post-discharge tele-bundle**	– A 2-month rehabilitation program based on the tailored rehabilitation plan established during the ward bundle, including nutritional guidance, swallowing rehabilitation, physical therapy, respiratory therapy, and psychological follow-up – Hand off the case to the primary care physician	– Rehabilitation specialists via centralized tele-rehabilitation through smartphone video conferencing	– Post-ICU rehabilitation has the potential to accelerate recovery, enhance functional outcomes, and improve the overall quality of life by addressing the physical, cognitive, and psychological challenges faced by critical care survivors

ICU - intensive care unit; RASS - Richmond Agitation Sedation Scale; SBT - spontaneous breathing trial; NRS - nutritional risk screening; EAT-10 - Eating Assessment Tool-10; HADS - Hospital Anxiety and Depression Scale.

* A critical care nurse navigator will participate in multidisciplinary discussions with the ICU staff, ward staff, and tele-rehabilitation clinicians to ensure continuity of care, address challenges, and provide ongoing guidance throughout the intervention phases;

✝ usual cutoffs will be used to screen patients at high risk for post-ICU complications (i.e., NRS 2002 > 2; EAT-10 > 2; Barthel index < 100; HADS anxiety or depression domains > 7.^(19-22)^

During the control phase, participating ICUs will be advised to maintain their standard care practices per local protocols.

### Implementation process

The following strategies will be employed to optimize adherence to the intervention:

– Formation of local implementation action teams: multidisciplinary teams composed of healthcare professionals will be established at each participating hospital to oversee and support the implementation process.
– Provision of educational materials: comprehensive educational materials, including written protocols and remote-based learning videos detailing the components of the study intervention, will be made available to all participating ICUs at the time of implementation.
– On-site training: the coordination center team will conduct on-site meetings with local staff (ICU and ward professionals) within 15 days before the intervention begins for training and once during the intervention phase (15 - 21 days after the intervention) for troubleshooting and re-training.
– Consultations with critical care specialists: local implementation teams will consult via telemedicine weekly with critical care specialists. These sessions will utilize evaluative and iterative approaches, such as Plan-Do-Study-Act (PDSA) cycles, to address challenges, refine processes, and ensure consistent adherence to the intervention. Additionally, critical care specialists will be available daily to provide support and answer any queries from the local teams.
– Virtual benchmarking meetings: virtual benchmarking meetings will be held 30 days (± 3 days) after the intervention begins. These meetings will involve hospitals randomized in the same sequence (to avoid contamination), offering a platform to address implementation challenges, foster peer learning, and encourage collaborative problem-solving.
– Patients eligible for tele-rehabilitation who do not have access to a smartphone will be provided with one to enable video conferencing.

### Fidelity of the program processes

Weekly remote monitoring visits will be conducted at all participating clusters to assess the fidelity of the proposed intervention. Researchers from the coordinating center will perform semi-structured interviews with ICU and ward staff to evaluate adherence to the recommended processes (see the interview template in the Supplementary Material). Additionally, patient participation rates will be measured as an indicator of adherence to the tele-rehabilitation component. Each cluster will be assessed during the intervention period using semi-structured evaluations, which will be scored from zero to 100%, with higher scores indicating greater adherence to the intervention. Adherence will be evaluated across three domains: ICU tele-bundle, including frequency of multidisciplinary rounds, analgesia optimization, sedation minimization, spontaneous breathing trials, delirium prevention, early mobilization, and removal of unnecessary invasive devices; ward tele-bundle, involving the screening of patients at high risk for post-ICU complications and the development of tailored rehabilitation plans during ward hospitalization; and post-discharge tele-bundle, assessed by the patient's attendance rate at teleconsultations. The overall adherence score will be calculated as the mean of the scores across all three domains.

### Outcomes

#### Primary outcome

The primary outcome is the health-related quality of life utility score measured 3 months after hospital discharge using the EuroQol five-dimension three-level (EQ-5D-3L) questionnaire.^(23)^ The EQ-5D-3L includes a descriptive system with five dimensions of health-related quality of life: mobility, self-care, usual activities, pain/discomfort, and anxiety/depression. Each dimension has three levels (no problems, some problems, and extreme problems). The utility score derived from the descriptive system for the Brazilian population ranges from −0.17 (where zero represents a health state equivalent to death; negative values are considered worse than death) to 1 (best health state). The estimated minimal clinically important difference for the EQ-5D-3L ranges from 0.03 to 0.52,^(24)^ with the mean value for the Brazilian population being 0.82.^(25)^ Deceased patients will be assigned a score of zero during follow-up, as mortality is a competing risk for quality of life assessment.

#### Secondary outcomes

Secondary outcomes include rehospitalization within 30 days after hospital discharge; all-cause mortality and clinical status according to a modified version of the World Health Organization Ordinal Scale for Clinical Improvement within 90 days^(26)^ (Table 1S - Supplementary Material); and the following outcomes measured at 90 days: days alive and free of hospital, symptoms of anxiety and depression assessed by the Hospital Anxiety and Depression Scale (with scores > 7 and > 10 indicating possible and probable cases of anxiety or depression, respectively),^(22)^ cognitive impairment assessed by the modified Telephone Interview for Cognitive Status (with scores < 32 indicating cognitive impairment),^(27)^ new disabilities in instrumental activities of daily living assessed by the Lawton and Brody instrumental activities of daily living scale^(28)^ (any impairment, moving from independent to partially dependent or from partially dependent to totally dependent, in at least one of the following domains: telephone use, transportation, shopping, responsibility for own medications, and ability to handle finances) relative to 1 month before hospitalisation, physical dependence assessed by the modified Barthel Index (with scores < 21, 21 - 60, and 61 - 90, indicating total, severe, and moderate dependence, respectively),^(29)^ EQ-5D-3L utility scores among survivors, and return to work or studies for patients who were employed or enrolled in education at the time of hospital admission.

### Follow-up

Trained site researchers will monitor participants daily during hospitalization, with remote supervision from coordinating center data managers and monitors. After discharge, centralized telephone interviews will be conducted by researchers blinded to the allocation group at 15, 30, 60, and 90 days for outcome assessment. The scheduled dates for these interviews will be relative to the day of hospital discharge (D0), with a ± 5-day window for the first follow-up (at 15 days) and a ± 15-day window for the subsequent follow-ups (at 30, 60, and 90 days).

### Procedures to ensure quality

Trained research personnel will prospectively collect data on printed case report forms, which will then be entered into an electronic data capture system (REDCap, Vanderbilt University, Tennessee, USA). To ensure adherence to the study protocol and data quality, the following procedures will be employed:

– All local principal investigators and subinvestigators will attend an on-site training session before the study's initiation to standardize procedures, including data collection methods, confidentiality procedures, and participant retention.
– Investigators will have access to the coordinating center for assistance in resolving any issues or concerns that may arise during the study.
– The coordinating center will review detailed weekly reports on screening, inclusion, follow-up, and data consistency and completeness. Immediate action will be taken to address any identified issues. Discrepancies - defined as improbable values potentially resulting from typos or data entry errors (e.g., an age of 110 years) - and missing data, will be identified through ongoing monitoring by the data management team. Participating centers will be promptly notified and asked to review and verify the accuracy of the flagged entries.

### Sample size

A minimum of 18 ICUs, each recruiting 100 patients (totaling 1,800 patients), will be required to detect a difference of 0.09 in EQ-5D-3L utilities (within the minimum clinically relevant difference)^24^ between the two study arms. This calculation assumes a power of 95%, a standard deviation of 0.28 for EQ-5D-3L utilities (estimated based on a previous publication),^(30)^ a cluster stepped-wedge design with four randomization sequences and five periods, an intra-cluster correlation coefficient of 0.05, and a two-sided alpha level of 0.05. To compensate for potential ICU and patient losses, the present study plans to recruit 20 ICUs.

### Statistical analysis

A detailed statistical analysis plan will be finalized, published, or made available online before locking the trial database and starting comparative data analysis. Missing values for the EQ-5D-3L and variables used for covariate adjustment will be imputed using multiple imputation by chained equations if the missing rate exceeds 5%. If the proportion of missing data is 5%, analyses will be conducted using complete cases only. Comparisons will be conducted at the participant level, accounting for cluster and period effects, with outcomes analyzed according to randomization group. Outcome comparisons between study arms will be performed using a mixed-effects generalized linear model, adjusted for age, gender, comorbidities, and baseline Simplified Acute Physiology Score III (SAPS III). Covariate adjustment is justified by the study design, as relevant baseline differences may persist despite a large sample size - especially given the potential for seasonal variations in case mix. Pre-planned sensitivity analyses for the primary outcome will assess the effects of the intervention based on quartiles of the overall cluster adherence score and adherence within each domain. There will be five *a priori* defined subgroup analyses for the primary outcome: age (< 65 years *versus* ≥ 65 years), sex, SAPS III score (< *versus* median), pre-admission Barthel index (< 90 *versus* ≥ 90), and COVID-19 *versus* other etiologies of acute respiratory failure. The consistency of intervention effects across the above-mentioned subgroups will be assessed through tests for interaction. A significance level of 0.05 will be applied to all statistical comparisons. Data analysis will be conducted using R (R Development Core Team).^(31)^

### Ethics and dissemination

#### Ethics approval and consent to participate

This study will be conducted according to resolution no. 466/12 of the Brazilian National Health Council^(32)^ and the Guidelines for Good Clinical Practice E6(R1).^(33)^ The institutional review board of the coordination center (CAAE: 70877623.8.1001.0071; approval number: 6.280.961) and all participating centers approved the study. A mixed consent process will be implemented. At the cluster level, written consent for the study protocol will be obtained from the ICU and ward head (cluster representative). At the participant level, the requirement for written informed consent was waived for hospital data, as the interventions focus on organizational aspects of care and do not involve untested clinical procedures. However, written consent will be required for post-ICU data collection and will be obtained from patients or their proxies at hospital discharge. If a patient or proxy refuses consent at hospital discharge, they will be asked whether the refusal applies to all data collection or only to the post-discharge period; the participant will be excluded accordingly.

#### Dissemination

We plan to make the study findings widely available and plan to disseminate our results in international conferences and peer-reviewed journals. Authorship will be determined according to the criteria of the International Committee of Medical Journal Editors.^(34)^ We plan to share data upon reasonable request.

### Discussion and trial status

**Figure 2:**
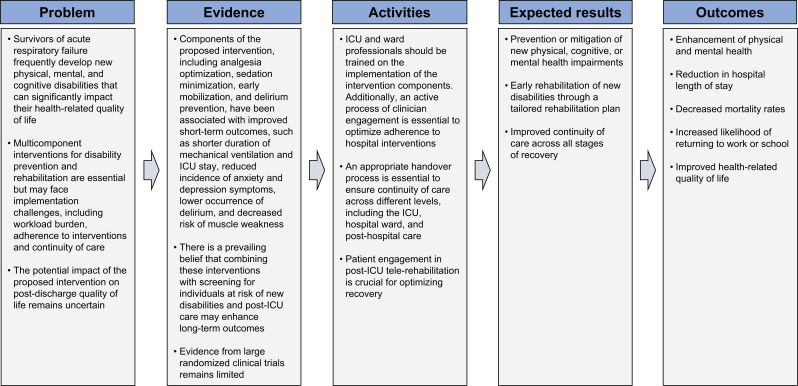
Logic model for multicomponent telemedicine-based intervention* on health-related quality of life of patients with respiratory failure requiring invasive mechanical ventilation.

Multicomponent interventions aimed at disability prevention and rehabilitation in patients with respiratory failure are complex, involving multiple interacting components.^(35)^Figure 2 presents the logic model underlying the intervention evaluated in this study. While several outcomes are expected to have a positive impact, we selected health-related quality of life as the primary outcome due to its strong potential for a causal and direct association and its significant clinical relevance. Reduced quality of life is a highly prevalent ICU complication, associated with increased burden for patients and their families, as well as higher healthcare costs.^(36–38)^ Therefore, identifying interventions that can mitigate the risk and burden of reduced quality of life in ICU patients is crucial for improving healthcare quality.

ICU - intensive care unit.

*The intervention consists of a quality improvement program focused on disability prevention and rehabilitation strategies, implemented during the patient's intensive care unit stay, continued through ward admission, and extending up to 3 months post-hospital discharge with tele-rehabilitation support.

Other important outcomes - such as all-cause mortality, rehospitalization, symptoms of anxiety and depression, cognitive impairment, and new disabilities affecting instrumental activities of daily living - may have both direct and indirect relationships with the proposed intervention.^(13,39–44)^ As such, they serve as key markers of its effectiveness and safety.

To the best of our knowledge, this will be the first large-scale, multicenter randomized trial evaluating the effects of a multicomponent telemedicine-based intervention on long-term health-related quality of life among survivors of acute respiratory failure. The study's strengths include an appropriate design for assessing quality improvement implementation, a large sample size, and using strategies recommended by complex intervention frameworks.^(35,45,46)^ These strategies include implementation optimization through the formation of local implementation action teams, provision of educational materials, consultations with critical care specialists, virtual benchmarking meetings, and an assessment of program fidelity. The results of this study will provide healthcare professionals, researchers, and policymakers with valuable insights into the efficacy and safety of bundled interventions for acute respiratory failure patients, particularly given the high scalability potential of telemedicine-based approaches.

Our study also has limitations. First, while the cluster stepped-wedge design ensures that all ICUs eventually receive the intervention, it is susceptible to biases such as time-dependent confounding (e.g., improvements in ICU care over time independent of the intervention and seasonality). Second, ensuring consistent adherence to protocol implementation across all clusters is challenging, given the multiple components, remote nature of the intervention, and diverse care settings the intervention targets (ICU, ward, and post-discharge). Variability in adherence and execution across sites could affect the intervention's consistency and effectiveness. Third, despite randomization, differences in patient severity, staffing levels, adherence to protocols, and institutional rehabilitation practices might influence outcomes. While the study likely adjusts for some variables, residual confounding remains a concern. Fourth, the EQ-5D-3L questionnaire is a widely used instrument for assessing health-related quality of life, but it may not fully capture all aspects of post-ICU disability. Assigning a utility score of zero to deceased patients is a reasonable approach to integrating mortality, but it may oversimplify the broader consequences of survival with severe disability. Fifth, follow-up assessments (e.g., 90-day EQ-5D-3L evaluations) may be challenging due to loss to follow-up, particularly in a critically ill population. Patients discharged to long-term care or those with severe disabilities may have higher attrition rates, introducing potential bias. Finally, the study is limited to ICUs in Brazil, which may impact the external validity of the findings. Differences in healthcare infrastructure, staffing ratios, and rehabilitation resources across regions and countries may limit the generalizability of the results.

The study design and protocol were finalized in September 2023, and all site investigators were trained. Currently, this study is recruiting subjects in 20 ICUs representative of the Brazilian geopolitical territory. We expect to complete follow-up by October 2025.

## Data Availability

After publication the data will be available on demand to authors.
