# Supplementary material for: The impact of a multicomponent telemedicine-based intervention on quality of life in adults with respiratory failure requiring mechanical ventilation: protocol for a cluster stepped-wedge randomized clinical trial (Tele-Rehab MV Trial)
Source: Crit Care Sci. 2025 Nov 27;37:e20250136. doi: 10.62675/2965-2774.20250136 (PMC12674818; doi:10.62675/2965-2774.20250136)
Supplement: Supplementary Material [file 2965-2774-ccsci-37-e20250136-suppl.pdf]

Critical Care Science
https://doi.org/10.62675/2965-2774.20250136

SUPPLEMENTARY MATERIAL

The impact of a multicomponent telemedicine-based intervention on quality of life in adults with respiratory failure requiring mechanical ventilation: protocol for a cluster stepped-wedge randomized clinical trial (Tele-Rehab MV Trial)

Adriano José Pereira*[1], Rafael Barberena Moraes*[2,3], Geraldine Trott[2], Maura Cristina dos Santos[1], Duane Mocellin[2], Alessandra Yuri Takehana de Andrade[1], Aline Paula Miozzo[2], Luisa de Castro Miranda Paixão[1], Raíne Fogliati de Carli Schardosin[2], Carla Luciana Batista[1], Emelyn de Souza Roldão[2], Cilene Saghabi de Medeiros Silva[1], Rosa da Rosa Minho dos Santos[2], Maria Isabel Costa e Silva Cavalcanti[1], Jennifer Menna Barreto de Souza[2], Luciana Diniz Nagem Janot de Matos[1], Denise de Souza[2], Juliana Wanderley Cidreira Neves[1], Gabriela Soares Rech[2], Thais Martins de Almeida Souza[1], Gabrielle Nunes da Silva[2], Carolina Rothmann Itaqui[2], Silvana Maria Silva Yoshida[1], Raquel Afonso Caserta Eid[1], Marcio Luiz Ferreira de Camillis[2], Kamilla Silvestre Rahman Genena[1], Leonardo Miguel Correa Garcia[2], Ester Cavalcanti Schaefer[1], Priscila Alves Pereira Cidade[2], Nara Fabiana Mariano[1], Isadora Rebolho Sisto[2], Ana Cristina Lagoeiro Patrocinio da Cruz[1], Camille Lacerda Corrêa[2], Ivan Ramos Maia[1], Juliana de Oliveira[2], Andrea de Carvalho[1], Marcio Ramos Laguna[2], Leonardo Rolim Ferraz[1], Cassiano Teixeira[2,3], Yasmin Ferreira Cavaliere[1], Fernando Godinho Zampieri*[1], Regis Goulart Rosa*[2,3]

* Contributed equally to the work.

SEMI-STRUCTURED INTERVIEW TEMPLATE TO BE PERFORMED WITH ICU STAFF.

Form 21 - Intensive care unit weekly adherence

INTENSIVE CARE UNIT BUNDLE ADHERENCE EVALUATION FORM			
Perceived weekly adherence of the center to daily multidisciplinary rounds	○ 10 rounds performed from Monday to Sunday (7 days a week)<br>○ 8 rounds performed only from Monday to Friday<br>○ 5 rounds performed 4 times or less per week		
Perceived daily adherence of the center to multidisciplinary rounds (patient level)	○ 100% of patients with acute respiratory failure included in the study were discussed in rounds;<br>○ 80 to 99% of patients with acute respiratory failure included in the study were discussed in rounds;<br>○ 60 to 79% of patients with acute respiratory failure included in the study were discussed in rounds;<br>○ 40 to 59% of patients with acute respiratory failure included in the study were discussed in rounds;<br>○ 20 to 39% of patients with acute respiratory failure included in the study were discussed in rounds;<br>○ 0 to 19% of patients with acute respiratory failure included in the study were discussed in rounds.		
Score:	______________________		
**PERCEPTION OF PERFORMING ADEQUATE ANALGESIA**			
Center adhered to the standardized pain assessment scales	○ Yes	○ Somewhat	○ No

Continue...

This is an open access article under the CC BY license (https://creativecommons.org/licenses/by/4.0/).

...continuation

Pain recorded in medical records according to routine vital signs	◯ Yes	◯ Somewhat	◯ No
Pre-emptive analgesia indicated	◯ Yes	◯ No	
**CONDUITS CARRIED OUT ACCORDING TO THE FLOWCHART**			
Evaluation of reversible causes of pain	◯ Yes	◯ Somewhat	◯ No
Nonpharmacological measures	◯ Yes	◯ Somewhat	◯ No
Pharmacological measures	◯ Yes	◯ Somewhat	◯ No
Reassessment of pain after management	◯ Yes	◯ No	
Final score of the conducts (Questions 3 and 3.1)	____________________		
**PERCEPTION OF MINIMIZING EXPOSURE TO SEDATION**			
Center adhered to sedation assessment using the RASS scale	◯ Yes	◯ Somewhat	◯ No
RASS recorded in patient medical records according to routine	◯ Yes	◯ Somewhat	◯ No
Sedation pause or titration performed for target RASS -2 to 0 in indicated patients	◯ Yes	◯ Somewhat	◯ No
Sedation reduction in patients with RASS < -2	◯ Yes	◯ Somewhat	◯ No
Evaluation of reversible causes of agitation for patients with RASS > 0	◯ Yes	◯ Somewhat	◯ No
Final score of the conducts (Question 4.1)	____________________		
**PERCEPTION OF PERFORMING THE SPONTANEOUS RESPIRATION TEST (SBT):**			
Center adhered to SBT condition evaluation	◯ Yes	◯ Somewhat	◯ No
Center performed SBT for the indicated cases	◯ Yes	◯ Somewhat	◯ No
Final score of spontaneous breathing	____________________		

Continue...

...continuation

Perceived adherence to *delirium* prevention:	○ 5 measures were implemented<br>○ 4 measures were implemented<br>○ 3 measures were implemented<br>○ Only 2 measures were implemented<br>○ Only 1 measure was implemented<br>○ No measures were implemented					
Final *delirium* prevention score	______________					
**PERCEPTION REGARDING PATIENT MOBILIZATION**						
Center evaluated the patients included in the study with the objective of mobilization	○	Yes	○	Somewhat	○	No
Center assessed the possibility of leaving the bed in the patients included in the study	○	Yes	○	Somewhat	○	No
Center mobilized eligible patients during the day	○	Yes	○	Somewhat	○	No
Eligible patients left their bed	○	Yes	○	Somewhat	○	No
Final patient mobilization score	______________					
**PERCEPTION REGARDING THE REMOVAL OF UNNECESSARY INVASIVE DEVICES**						
Center adhered to the invasive device removal evaluation	○	Yes	○	Somewhat	○	No
Center immediately removed the indicated invasive devices	○	Yes	○	Somewhat	○	No
Final score of unnecessary invasive devices	______________					
Perception of adherence to training and continuing education for bundle implementation	○ 100% of the team was trained<br>○ 80 to 99% of the team was trained<br>○ 60 to 79% of the team was trained<br>○ 40 to 59% of the team was trained<br>○ 20 to 39% of the team was trained<br>○ 0 to 19% of the team was trained					
Final adherence score (review)	______________					
Perception regarding team engagement	1 [ ][ ][ ][ ][ ][ ][ ][ ][ ][ ] 10<br>*(Place a mark on the scale above)*					
Comments on the contact (strengths and weaknesses)	______________					

Table 1S - Modified ordinal scale for clinical improvement

1. At home without needing assistance for instrumental activities of daily living (e.g., feeding, hygiene, mobility, medication management)
2. At home but requiring assistance for instrumental activities of daily living.
3. Hospitalized in the ward and ambulating.
4. Hospitalized in the ward, able to sit unassisted but not ambulating.
5. Hospitalized in the ward and bedridden
6. Hospitalized in the intensive care unit, not on invasive mechanical ventilation, and able to sit
7. Hospitalized in the intensive care unit, not on invasive mechanical ventilation, but unable to sit
8. Hospitalized in the intensive care unit and on invasive mechanical ventilation
